# Newly discovered population of *Aedes japonicus japonicus* (Diptera: Culicidae) in Upper Bavaria, Germany, and Salzburg, Austria, is closely related to the Austrian/Slovenian bush mosquito population

**DOI:** 10.1186/s13071-016-1447-z

**Published:** 2016-03-21

**Authors:** Dorothee E. Zielke, Doreen Walther, Helge Kampen

**Affiliations:** Leibniz-Centre for Agricultural Landscape Research, Eberswalder Str. 84, 15374 Muencheberg, Germany; Friedrich-Loeffler-Institut, Federal Research Institute for Animal Health, Suedufer 10, 17493 Greifswald – Insel Riems, Germany

**Keywords:** *Aedes japonicus japonicus*, Asian bush mosquito, Austria, Germany, Human-mediated displacement, Microsatellites, *nad*4 haplotypes, Population genetics, Spread

## Abstract

**Background:**

The German mosquito surveillance instrument ‘Mueckenatlas’ requests the general public to collect and submit mosquito specimens. Among these, increasing numbers of individuals of invasive species have been registered. Specimens of the Asian bush mosquito *Aedes japonicus japonicus* submitted from German Upper Bavaria, where this species had not previously been recorded, triggered regional monitoring in mid-2015.

**Methods:**

The search for *Ae. j. japonicus* breeding sites and developmental stages concentrated on cemeteries in the municipality of origin of the submitted specimens and, subsequently, in the whole region. A virtual grid consisting of 10 × 10 km^2^ cells in which up to three cemeteries were checked, was laid over the region. A cell was considered positive as soon as *Ae. j. japonicus* larvae were detected, and regarded negative when no larvae could be found in any of the cemeteries inspected. All cells surrounding a positive cell were screened accordingly. A subset of collected *Aedes j. japonicus* specimens was subjected to microsatellite and *nad*4 sequence analyses, and obtained data were compared to individuals from previously discovered European populations.

**Results:**

Based on the grid cells, an area of approximately 900 km^2^ was populated by *Ae. j. japonicus* in Upper Bavaria and neighbouring Austria. Genetic analyses of microsatellites and *nad*4 gene sequences generated one genotype out of two previously described for Europe and three haplotypes, one of which had previously been found in Europe only in *Ae. j. japonicus* samples from a population in East Austria and Slovenia. The genetic analysis suggests the new population is closely related to the Austrian/Slovenian population.

**Conclusion:**

As *Ae. j. japonicus* is well adapted to temperate climates, it has a strong tendency to expand and to colonise new territories in Central Europe, which is facilitated by human-mediated, passive transportation. The new population in Upper Bavaria/Austria is the seventh separate population described in Europe. According to our data, it originated from a previously detected population in eastern Austria/Slovenia and not from an introduction event from abroad. The dispersal and population dynamics of *Ae. j. japonicus* should be thoroughly surveyed, as this species is a potential vector of disease agents.

**Electronic supplementary material:**

The online version of this article (doi:10.1186/s13071-016-1447-z) contains supplementary material, which is available to authorized users.

## Background

**Fig. 1:**
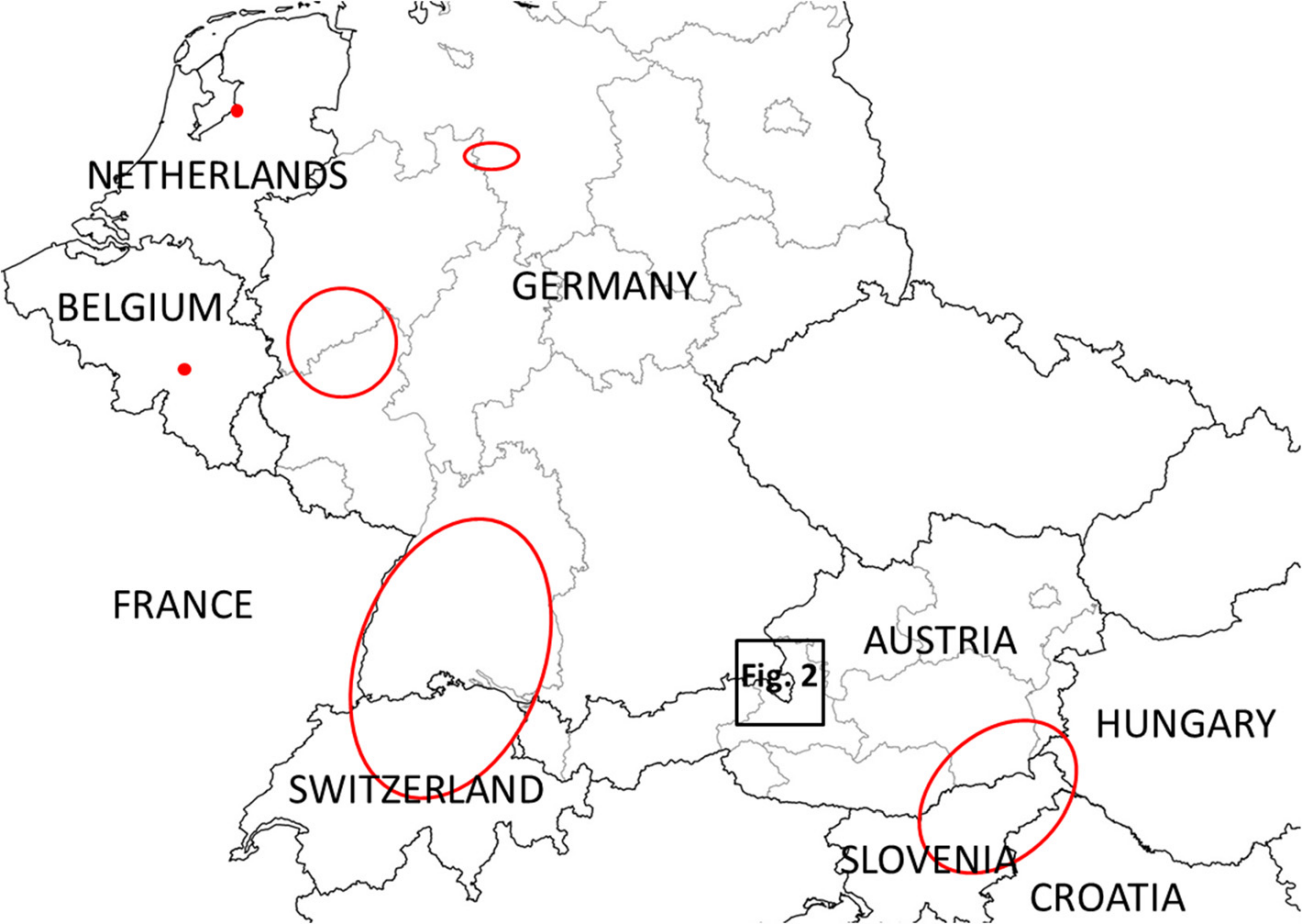
Geographical location of previously detected *Ae. j. japonicus* populations in Europe (red dots and circles) and of the newly discovered population (Fig. 2 inset)

The invasive Asian bush mosquito *Aedes* (*Hulecoeteomyia*) *japonicus japonicus* (Theobald, 1901) (taxonomic nomenclature according to [1]) was first reported from Europe in 2000 when it was detected in Normandy (Orne Department) in northern France [2]. While the species was later eliminated there [3], another population detected in 2002 in Belgium succeeded in establishing [4]. After more than a decade of remaining locally restricted, it was eventually controlled and is now also considered eliminated (pers. comm. Versteirt 2016). Several additional populated areas were discovered in Europe between 2008 and 2014 (Fig. 1): Switzerland/Southwest Germany/France [3, 5, 6], west Germany [7], north Germany [8], Austria/Slovenia/Croatia/Hungary [9–11] and The Netherlands [12].

*Aedes j. japonicus* is native to east Asia (Japan, Korea, southern China, southeastern Russia) where it colonises regions climatically similar to central Europe [13]. It has been transported intercontinentally by air and sea since the early 1990s and was demonstrated as invasive in the late 1990s in the US where it is now widely distributed [14].

*Aedes j. japonicus* is a potential vector of disease agents, able to transmit Getah, Japanese encephalitis, West Nile, dengue, chikungunya and Rift Valley fever viruses in the laboratory [15–19]. In the field, it has been found infected with Japanese encephalitis, West Nile and La Crosse viruses [20–22].

After decades of neglect, the occurrence and spatiotemporal distribution of culicid species, including invasive ones such as *Ae. j. japonicus*, have been thoroughly monitored in Germany since 2011. In addition to active data sampling by trapping, a passive approach was implemented in 2012 in the form of a citizen-science project. The ‘Mueckenatlas’ (mosquito atlas) calls upon private persons to collect mosquitoes and submit them for mapping spatiotemporal mosquito occurrence [23]. Since its launch, it has contributed thousands of distribution records and has been particularly valuable regarding invasive mosquito species. It enabled the detection of the west and north German populations of *Ae. j. japonicus* in 2012 and 2013 [7, 8], of a reproductive population of the Asian tiger mosquito *Ae. albopictus* in south Germany in 2014 [24] and of the introduction of *Ae. koreicus* in 2015 [25].

In early July 2015, three adult specimens of *Ae. j. japonicus* were submitted to the ‘Mueckenatlas’ team from Berchtesgaden, a town in Upper Bavaria, southeastern Germany, immediately on the border with Austria. Since this region was far from areas of previous *Ae. j. japonicus* documentation, the submissions prompted immediate local monitoring.

## Methods

### Mosquito collection

On the occasion of a visit to Upper Bavaria (inset in Fig. 1) in August 2015, water containers on the premises and surroundings of the submitter were checked for *Ae. j. japonicus* immature stages. Larvae were found in water accumulated in the inverted top of a rain-water barrel in the garden of the submitter and in a bucket filled with debris and water in the neighbour’s garden. An inspection of the municipality’s cemetery, the distance to which was 1.3 km (direct line), also revealed *Ae. j. japonicus* larvae in many of the numerous water containers.

**Fig. 2:**
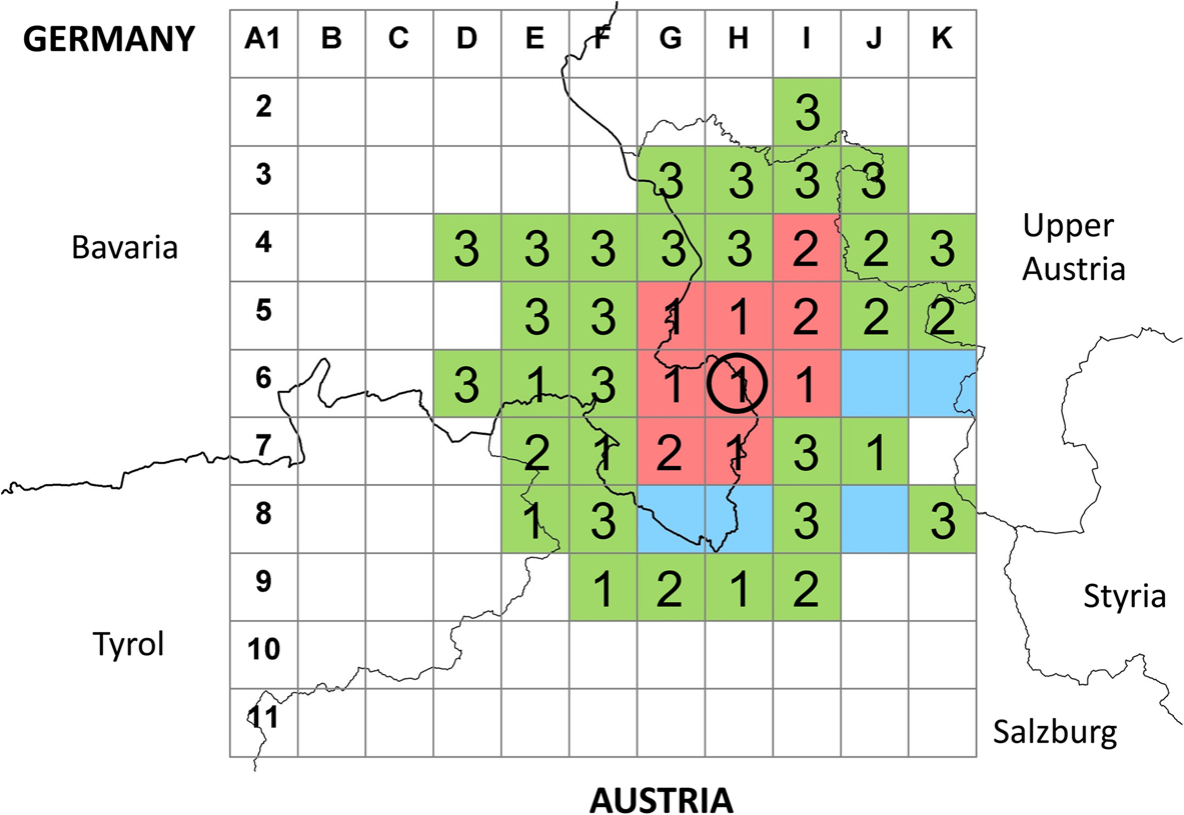
Area coverage of the Upper Bavarian/Austrian *Ae. j. japonicus* population (red squares: grid cells positive; green squares: grid cells negative by cemetery inspection; blue cells: not sampled due to mountainous settings without human settlements; figures in green cells: number of cemeteries identified and examined; figures in red cells: number of cemeteries examined until finding of *Ae. j. japonicus*; black circle: grid cell with *Ae. j. japonicus* submissions to the ‘Mueckenatlas’)

To gain an impression of the size of the populated area, a virtual grid with 10 × 10 km cells was laid over the region (Fig. 2). Starting in Berchtesgaden, all cells surrounding *Ae. j. japonicus*-positive cells were checked by inspecting water containers in cemeteries, as suggested by Vezzani [26] and demonstrated to be efficient by various other authors (e.g. [3, 6]). In smaller cemeteries all natural and artificial water-holding containers identified were examined, while in larger cemeteries the inspection was limited in time to one hour. Cells were considered positive as soon as *Ae. j. japonicus* larvae were found, irrespective of the number of cemeteries screened. Cells were rated negative if no larvae could be found in the cemeteries of three villages (provided three villages were present in the cell), or in one or two cemeteries in some more alpine and little-populated cells when no further cemeteries could be located.

*Aedes j. japonicus* larvae were identified on the spot by their habitus and behaviour (cf. [6]) but samples from each collection site were taken to the laboratory for further development until adult emergence and genetic analyses. Adults were identified morphologically according to the key by Schaffner *et al*. [27]. For confirmation, at least one specimen per site was identified genetically by CO1 DNA barcoding [12].

### Genetic analysis

To unveil putative relationships to other populations of *Ae. j. japonicus* in Europe, and to identify possible source populations, population genetic analyses, using *nad*4 (NADH dehydrogenase subunit 4) gene polymorphisms, were carried out on 30 specimens from the colonised area, according to protocols presented by Fonseca *et al*. [28] and Zielke *et al*. [29]. The same 30 individuals were subjected to analysis of seven microsatellite loci, with the results interpreted with GeneMapper (Applied Biosystems/Thermo Fisher Scientific, Waltham, MA, USA), STRUCTURE [30], STRUCTURE HARVESTER [31] and GenAlEx [32]. This approach had been specifically adapted and optimized to *Ae. j. japonicus* population genetics in previous work and has been proven effective [28, 29, 33, 34].

For DNA extraction, legs of adult mosquitoes or three to four abdominal segments of larvae were processed using the QIAamp DNA Mini Kit (Qiagen, Hilden, Germany), according to the manufacturer’s instructions.

## Results

*Aedes j. japonicus* immature specimens were found in nine grid cells, corresponding to an infested area of about 900 km^2^ stretching from Upper Bavaria to the Austrian federal state of Salzburg (Fig. 2). Of the nine grid cells positive, *Ae. j. japonicus* was found in the first cemetery checked in six cells, while in three cells the second cemetery checked was populated.

Among the analysed individuals (one individual was not analysable), three *nad*4 haplotypes were found: the ubiquitous H1 (5 individuals), as well as H9 (10 individuals) and H10 (14 individuals) (naming of haplotypes according to [34]), the latter previously described only in the population occurring in southeastern Austria and Slovenia [33].

To comparatively analyse the microsatellite data of the newly discovered Upper Bavarian/Austrian population, data of formerly examined specimens from populations found in Switzerland/southwest Germany, Belgium, west Germany, north Germany, The Netherlands and Austria/Slovenia [29, 33] were included (Table 1). A check for Hardy-Weinberg equilibrium showed significant deviations on locus OJ10 in the Upper Bavarian/Austrian population, due both to higher and to lower than expected heterozygosity.

**Table 1:** Number of specimens and determined genotypes of other European *Ae. j. japonicus* populations comparatively examined by microsatellite analyses

Population	Number of individuals	Principal microsatellite genotype	Reference
Austria/Slovenia	36/60	1	[29, 33]
Belgium	18	1	[29]
West Germany	197	2	[29]
North Germany	32	2	[33]
Switzerland/Southwest Germany	22/80	1, 2	[29, 33]
Netherlands	43	1, 2	[33]

**Fig. 3:**
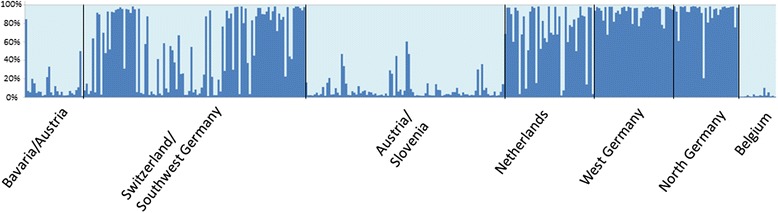
Bar plot of a Bayesian cluster analysis done with STRUCTURE [30] (light blue = genotype 1, dark blue = genotype 2)

The microsatellite data were further subjected to a Bayesian cluster analysis with the programme STRUCTURE [30], demonstrating genotype 1 for the Upper Bavarian/Austrian population out of two genotypes occurring in Europe (Fig. 3), as identified as the optimal number of genetic clusters for the complete dataset by STRUCTURE HARVESTER [31].

**Fig. 4:**
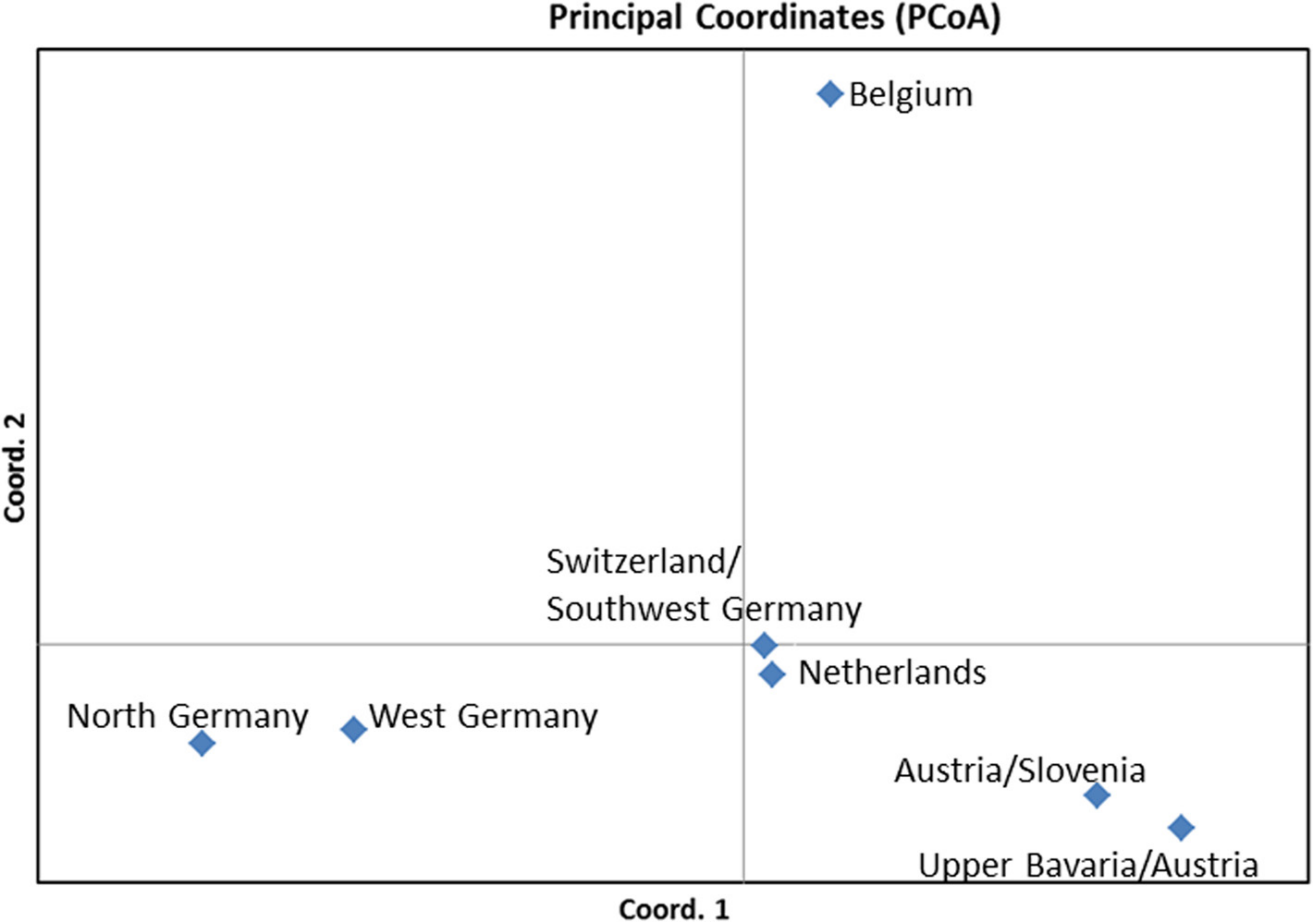
Principal coordinates analysis plot of pairwise population values of Nei’s genetic distance for the seven European *Ae. j. japonicus* populations

A principal coordinates analysis, based on a pairwise population matrix of Nei’s genetic distances (Additional file 1) and performed with GenAlEx [32] on the microsatellite data, shows a close genetic proximity of the newly discovered Upper Bavarian/Austrian population to the formerly analysed Austrian/Slovenian population (Fig. 4). The plot shows four groups of *Ae. j. japonicus* populations in Europe: The Belgian population is on top of a triangle with roughly the same genetic distance to the north and west German populations in one corner and to the Austrian/Slovenian and the Upper Bavarian/Austrian populations in the other corner. The Swiss/southwest German and Dutch populations, which are genetically more heterogeneous than the others (cf. [33]), are located between the north and west German populations on the one side and the Austrian/Slovenian and the Upper Bavarian/Austrian populations on the other side (Fig. 4).

## Discussion

A fourth German population of *Ae. j. japonicus*, representing the seventh European population of this species, was detected in southeastern Germany crossing the border to Austria. The distribution of this isolated population was roughly determined by a grid cell pattern with findings of immature stages of the species in some cells surrounded by cells without findings. This approach can serve merely as an indication of the extent of the population, as only cemeteries were inspected in the cells, and of these, only three per cell. Neither natural potential breeding sites, e.g. tree holes in forests, nor artificial breeding sites in gardens were checked.

No estimate is currently possible for the time of establishment of the Upper Bavarian/Austrian population. Its assumed distribution area is comparable in size to that of the north German population, which is supposed to be a relatively recent offshoot of the west German population [33] but has existed at least since 2012 [8]. Due to the area coverage, the Upper Bavarian/Austrian population might be of similar age, although a BG Sentinel trap operated in the affected area of Upper Bavaria from 2011 to 2013 (mid-April to late October each year once a week for 24 hours) never collected an *Ae. j. japonicus* specimen. The BG Sentinel is not particularly effective at collecting *Ae. j. japonicus* [35], but had already shown this species to occur in another German region previously not known to be populated (Werner & Kampen, unpubl.).

Climatic conditions are rather different in Upper Bavaria and northern Germany. German Upper Bavaria and the Austrian federal state of Salzburg are located at the northern boundaries of the Alps (minimum regional altitudes *c*. 400 m a.s.l.) and are characterised by a rather low annual average temperature (*c*. 7–8 °C) and monthly average temperatures exceeding 10 °C only from May to September. Snowfall typically occurs from early November to late April. Now that the distribution area of the *Ae. j. japonicus* population in Upper Bavaria/Austria has been determined, its future development and spread in this short-seasoned region should be directly compared with the north German population.

The *nad*4-haplotypes characterising the newly discovered Upper Bavarian/Austrian population suggest descent from the formerly detected Austrian/Slovenian population. Haplotype H9 is also known from the Belgian and the Dutch populations, but neither H9 nor H10 occur in any of the other German populations of *Ae. j. japonicus*. H10 had previously been demonstrated exclusively in the Austrian/Slovenian population in Europe. Outside of Europe, this haplotype has so far been described only from populations in New York and Connecticut in the USA [28], although it probably also exists in Asia where few studies have been made. With the available data, non-European source populations of the Upper Bavarian, Austrian and Slovenian individuals, therefore, cannot be narrowed down.

Microsatellite analyses show that the Upper Bavarian/Austrian population is assignable to genotype 1, the first *Ae. j. japonicus* genotype detected in Europe [29], although one individual suggests admixture with genotype 2. A close relationship with the Austrian/Slovenian population is shown, underlining the *nad*4 haplotype results. Conversely, the Upper Bavarian/Austrian population does not seem to be closely related to or to have the same origin as one of the other German populations.

## Conclusions

As the distribution area of the newly detected *Ae. j. japonicus* population in Upper Bavaria/Austria is beyond the flight distance to the population in east Austria, human-mediated transport of founder individuals, such as by eggs attached to used tyres, must be postulated. The Austrian Tauern Autobahn (motorway A10), which, coming from Slovenia, passes the Upper Bavarian/Austrian distribution area of *Ae. j. japonicus*, might be a possible route for the displacement of mosquitoes from east Austria or Slovenia into the Austrian federal state of Salzburg.

Given the high ecological plasticity of *Ae. j. japonicus*, more populations should be expected to emerge in subsequent years, with spatial coverage of large parts of central Europe.

## Additional file

Additional file 1: Pairwise population matrix of Nei’s genetic distances. The lower the values, the closer the genetic relatedness between the respective populations. (XLSX 9 kb)

## Abbreviations

CO1: cytochrome c oxidase subunit 1
*nad*4: NADH dehydrogenase subunit 4
